# Linking hydraulic strategy, drought response and carbon gain in co-existing savanna tree species

**DOI:** 10.1093/treephys/tpag024

**Published:** 2026-02-20

**Authors:** Benjamin J Wigley, Pierre-André Waite, Corli Coetsee, Bernhard Schuldt, Steven I Higgins

**Affiliations:** Plant Ecology, University of Bayreuth, Universitaetsstrasse 30, Bayreuth 95447, Bayern, Germany; School of Natural Resource Management, Nelson Mandela University, George Campus, Madiba Drive, George 6530, South Africa; Savanna Node, Scientific Services, SANParks, Skukuza 1350, South Africa; Cirad, Centre de coopération Internationale en Recherche Agronomique pour le Développement, UPR AIDA, Nairobi 00100, Kenya; Agroécologie et Intensification Durable des cultures Annuelles, Université de Montpellier, CIRAD, Avenue Agropolis, Montpellier, France; Department of Water and Agricultural Resource Management, University of Embu, Embu 60100, Kenya; School of Natural Resource Management, Nelson Mandela University, George Campus, Madiba Drive, George 6530, South Africa; Savanna Node, Scientific Services, SANParks, Skukuza 1350, South Africa; Chair of Forest Botany, TUD Dresden University of Technology, Pienner Straße 7, Tharandt 01737, Sachsen, Germany; Plant Ecology, University of Bayreuth, Universitaetsstrasse 30, Bayreuth 95447, Bayern, Germany

**Keywords:** drought avoidance, drought tolerance, growth, hydraulic traits, photosynthesis, safety margin–related traits, water potentials

## Abstract

Savannas cover a significant portion of the earth’s land surface, yet how they will respond to increases in rainfall variability and drought frequency and intensity expected with climate change remains poorly understood. Studies of hydraulic-related traits of savanna trees are rare with most existing research focusing on temperate and tropical forest species. We measured growth, photosynthetic rates, monthly predawn and midday xylem pressure potentials, and eight traits relevant to xylem, leaf safety and water storage capacity, in six co-occurring Southern African semi-arid savanna species. The six species adopted different hydraulic strategies, ranging from drought tolerance (e.g., high wood density, low xylem vulnerability to cavitation and ${{\psi}}_{{tlp}}$) in *Dichrostachys cinerea* (L.) Wight & Arn. to drought avoidance (e.g., high capacitance and shoot saturated water content) in *Terminalia sericea* Burch. ex DC. Drought-avoiding species with high capacitance had higher growth and photosynthetic rates, while drought-tolerant species had slow growth and low photosynthetic rates, when soil water was not limiting. The different hydraulic strategies found in the six study species suggest that savanna tree species exploit different ecohydrological niches, likely contributing to their co-existence in an environment where rainfall and soil water availability are highly variable. All of the strategies allowed for survival during shorter-growing season droughts. Previous studies have shown that both drought avoiders and tolerators may be vulnerable to mortality during more extensive droughts in savannas. We suggest that access to deeper soil water combined with higher capacitance, as found in *Sclerocarya birrea* (A.Rich.) Hochst. appears to be the most successful strategy to survive extensive drought.

## Introduction

Tree mortality is increasing in many regions of the world (Yi et al. 2022). While the mechanisms of tree mortality are not fully understood, increasing mortality rates appear to be correlated with increasing droughts that are associated with a warming and drying atmosphere (Sankaran 2019, McDowell et al. 2022, Yi et al. 2022). Both climate simulations and direct observations have shown an increase in precipitation intensity and rainfall variability over the last century for terrestrial ecosystems worldwide (Donat et al. 2016, Masson-Delmotte et al. 2021). This suggests a decrease in the number of rain days and an increase in the average rainfall amount per rain day, which result in longer dry periods between rain events, even if total rainfall remains unchanged (Sillmann et al. 2013, Bao et al. 2017, Pohl et al. 2017, Dunning et al. 2018).

The global trend of increasing tree mortality has resulted in an increased number of studies that predict drought-related tree mortality (McDowell et al. 2008, West et al. 2012, Rosas et al. 2019). Tree responses to drought have been interpreted through several complementary mechanisms. Tree survival may depend on the inherent resilience of the hydraulic transport system to declining water availability (Mitchell et al. 2013, Anderegg et al. 2015*a*, 2016, Zhang et al. 2019). The failure of the hydraulic system can also interact with other physiological stress pathways, such as carbon starvation and susceptibility to biotic agents, thereby compounding drought-induced mortality (McDowell et al. 2008, 2011, Anderegg et al. 2015*b*). In addition, hydraulic traits frequently emerge as important predictors of vulnerability under water-limited conditions (Sala et al. 2010, McDowell et al. 2011, Adams et al. 2017, Choat et al. 2018). These mechanisms interact in turn with species identity, plant size, rooting depth, resprouting ability and tree density, as well as topography and local edaphic features; e.g., whether trees are in wetter or cooler locations (Sankaran 2019, Trugman et al. 2021).

A biophysical reality of CO_2_ uptake during photosynthesis is the loss of water vapour to the atmosphere. When soil water is limited, high stomatal conductance (g${}_{{s}}$) results in increasingly negative leaf and stem water potentials. The greater tension in the water column that results from more negative water potentials eventually leads to cavitation within the xylem that can result in hydraulic failure (Sperry et al. 1993). Plants have evolved different hydraulic strategies to deal with water stress. Tardieu and Simonneau (1998) for example distinguished between isohydric and anisohydric strategies of water potential regulation. Plants adopting anisohydric strategies tolerate drought by leaving the stomata open for longer during drought and exposing their xylem to more extreme water potentials. Plants adopting isohydric strategies avoid experiencing drought by maintaining relatively stable water potentials during drought by closing the stomata. However, Hochberg et al. (2018) pointed out shortcomings in this dichotomous classification of drought strategies. For example, the same species can exhibit large variations in isohydricity, suggesting that species fall on a continuum, rather than in two distinct groups. Further recent work has shown that this dichotomy oversimplifies plant water-use behaviour, which instead varies continuously across species and contexts and depends on interacting traits related to hydraulics, stomatal regulation, carbon demand and drought history (Martínez-Vilalta and Garcia-Forner 2017, Kannenberg et al. 2022). Plant responses also shift with environmental conditions and timescales, making fixed isohydric or anisohydric labels insufficient for capturing the dynamic and multidimensional nature of plant water-use strategies (Martínez-Vilalta and Garcia-Forner 2017, Kannenberg et al. 2022). This limitation has prompted a shift toward functional groupings of drought strategies based on multiple plant traits and vulnerabilities, which may better capture variation in drought responses (Hochberg et al. 2018, Hood et al. 2018).

Tropical savannas are characterized by highly seasonal rainfall, typically with wet summer seasons and pronounced dry winter seasons ranging anywhere from 3 to 8 months (Scholes and Walker 1993, Duff et al. 1997, Bustamante et al. 2012). Temperatures remain high for most of the year, with the result that most savannas experience high vapour pressure deficits for extended periods (Duff et al. 1997, Scholes and Walker 2004, Goldstein et al. 2008). In addition, inter-annual rainfall variability is also a defining feature of the savanna biome, typically increasing with decreasing mean annual rainfall (Fensham et al. 2009). Thus, multiple consecutive years with below-average rainfall are not uncommon in most savannas (Fensham et al. 2009). The high levels of rainfall variability and frequent droughts, which interact with disturbances such as fire, are expected to result in a diverse array of coping mechanisms among tree species. Current understanding of functional groupings in savanna tree responses to drought remains limited, largely because most research on drought-induced tree mortality has focused on forest, thicket and Mediterranean ecosystems (Tardieu and Simonneau 1998, Choat et al. 2012, Anderegg et al. 2016, 2018, Adams et al. 2017, O’Brien et al. 2017, Aleixo et al. 2019, Skelton et al. 2025). Sankaran (2019) pointed out the paucity of hydraulic- and safety margin–related traits for savanna trees and proposed a list of tolerance traits and avoidance traits that will influence the growth and survival during drought, i.e., together conferring resistance. If species have hydraulic strategies that allow them to survive extended dry periods and effective carbon gain strategies to ensure that they can grow fast and reproduce during wetter periods, they should be better adapted to highly variable water availability (Franco 2009). A trade-off between fast growth and susceptibility to drought is expected, as this pattern represents a well-established life-history trade-off between growth maximization under optimal conditions and survival under environmental stress (Stearns 1989, Wright et al. 2010).

Likewise, because carbon gain and water loss are tightly coupled via stomatal conductance, leaf economic traits are linked to plant hydraulic function (Kröber et al. 2014). The leaf economic spectrum represents the general trade-off between resource-acquisitive leaves, which have high specific leaf area (SLA) and high photosynthetic capacity, and resource-conserving leaves that are long-lived but less photosynthetically efficient. Leaves with high SLA and high assimilation may facilitate rapid growth but might also need conductive xylem to support the ample water needs, which may be at the cost of being more prone to cavitation. Conversely, leaves at the other end of the spectrum tend to be thicker and lignified with less residual conductance, often pairing with drought-resistant hydraulic traits [e.g., high wood density (WD) and xylem with a higher resistance to embolism; Kröber et al. 2014].

In tropical savannas where hydraulic constraints are especially high, trait coordination across organs is most likely determinant for species survival. Such constraints drive two contrasting strategies. On one side, drought-deciduous species (often with higher SLA and peak photosynthetic rates) exploit favourable wet-season conditions for maximal carbon gain, then shed leaves as dry-season vapour pressure deficit rises. On the opposite side, evergreen species maintain foliage year-round but at a lower gas-exchange rate, relying on tough, low-SLA leaves and more resistant xylem (Chen et al. 2021). Nonetheless, despite an avoidance of the dry season, deciduous trees may suffer greater canopy die-back and mortality in severe droughts than evergreen species, which have more conservative hydraulic strategies (e.g., more resistant xylem, tighter stomatal control and greater investment in hydraulic safety) (Chen et al. 2021), especially in a context of more intense climate change droughts.

Here, for six dominant Southern African savanna tree species (Table 1), we have used a comprehensive set of hydraulic traits (Table 2), photosynthesis and growth rate measurements to determine if distinct hydraulic and growth strategies or syndromes exist among co-occurring species. Tolerance traits include the water potential at which 50% xylem hydraulic conductivity is lost as a result of xylem embolism, i.e., cavitation resistance (*P*_50_), leaf turgor loss point (Ψtlp, leaf water potential at which wilting occurs), the residual stomatal conductance residual stomatal conductance when stomata are closed (${{g}}_{{min}}$), WD and SLA. Avoidance traits include hydraulic capacitance and high saturated water content (i.e., internal water storage). In addition, we propose that relative water content at the key threshold at leaf turgor loss point (RWCtlp) will give a useful indication of the plant’s tendency to conserve water when stomata are closed, as well as the water reserves available to buffer against drought. We additionally measured parameters indicating plant water status (${{\psi}}_{{pd}}$ and ${{\psi}}_{{md}},$ which are the xylem pressure potentials at predawn and midday, respectively), ecological performance under optimal conditions (peak diameter growth rate and annual diameter increment) and photosynthetic rates (maximum carboxylation rate of Rubisco, maximum rate of net photosynthesis and rate of electron transport at maximum light intensity).

**Table 1:** The selected study species and their characteristics

Species	Average height (m)	Broad/fine-leaved	Deep/shallow rooted	Winner/loser
*Combretum apiculatum* Sond.	5	Broad-leaved	Shallow	Loser
*Acacia nigrescens* Oliv.	8	Fine-leaved	Intermediate	Winner
*Sclerocarya birrea* (A. Rich.) Hochst.	10	Broad-leaved	Deep	Winner
*Terminalia sericea* Burch. ex DC.	6	Broad-leaved	Shallow	Loser
*Dichrostachys cinerea* (L.) Wight & Arn.	3	Fine-leaved	Shallow	Loser
*Cassia abbreviata* Oliv.	6	Broad-leaved	Deep	Winner

Rooting depth is based on Zhou et al. (2020) and winner/loser is an indication of drought tolerance based on Case et al. (2020) and Swemmer (2020).

**Table 2:** Summary of investigated traits

Type	Trait	Abbreviation	Unit	Significance
Growth indicators	Annual diameter increment	ADI	mm	Indicator of overall tree productivity and performance
	Peak diameter growth rate	PDGR	mm year^−1^	Reflects the potential maximum growth rate
	Specific leaf area	SLA	cm^2^ g^−1^	Relates to resource allocation and light interception efficiency
Photosynthetic performance indicators	Maximum carboxylation rate of Rubisco	*V_cmax_*	μmol m^2^ s^−1^	Determines photosynthetic capacity and relates to plant productivity
	Rate of electron transport at 2000 μmol m^2^ s^−1^ photon flux density	*J_max_*	μmol m^2^ s^−1^	Reflects electron transport capacity and relates to photosynthetic rates
	Maximum rate of net photosynthesis	*A_max_*	μmol m^2^ s^−1^	Represents peak photosynthetic rate and re- lates to plant productivity
Water relation indicators	Xylem pressure potentials at predawn (3 a.m. to 6 a.m.) and midday (11 a.m. to 1 p.m.)	*ψ_pd_* and *ψ_md_*	MPa	Indicates least negative and most negative water potential experienced by a plant within a day
	Whole shoot saturated water content (i.e., max amount of water per dry mass)	SWC*_br_*	g g^−1^	Indicates water storage capacity in above-ground parts; relates to a plant’s ability to buffer against drought
	Relative water content (i.e., amount of water per max amount of water) at ψ_tlp_	RWC*_tlp_*	g g^−1^	Indicates remaining water in the shoot at leaf wilting point; relates to plant strategies to conserve water
	Hydraulic capacitance	C*_std_*	kg^−1^ MPa^−1^	Ability to store water in tissues and gradually release it during increasing drought stress
	Leaf residual conductance	*g_min_*	mmol m^−2^ s^−1^	Residual conductance after stomatal closure; relates to ability of plants to minimize water leakage during increasing drought stress
	Water potential at 50% loss of hydraulic conductivity	*P*_50_	MPa	Relates to xylem safety during a drought
	Leaf water potential at turgor loss point	ψ*_tlp_*	MPa	Related to leaf safety during a drought (turgor loss = wilting point)
	Wood density	WD	g cm^−3^	Relates to resource allocation, mechanical support and hydraulic efficiency

We indicated their type (i.e., the general category of each trait), symbols and units, as well as the significance of each trait when used to characterize tree ecological strategies.

In savanna trees, we expect the need to balance carbon acquisition with water conservation to result in a set of complex interactions between photosynthesis, growth and drought tolerance. Disruptions in water transport reduce stomatal conductance and CO_2_ uptake, directly limiting photosynthetic rates and growth (Hubbard et al. 2001, Brodribb and Holbrook 2007). We aim to investigate the presence of a trade-off between drought tolerance and performance during optimal conditions (growth/photosynthetic rates) in savanna woody plants, and to assess whether variation among species is best described as a continuum rather than discrete strategies. We propose that species with high photosynthetic rates and rapid growth should exhibit traits that enhance water uptake and utilization efficiency, which should support vigorous growth during favourable conditions. However, these traits can also make trees more vulnerable to drought-induced cavitation. Conversely, species with higher drought tolerance are expected to exhibit traits that reduce water loss and enhance water storage, which also often result in lower photosynthetic rates and slower growth, as they prioritize water conservation over carbon acquisition.

## Materials and methods

### Study site and species descriptions

The study was undertaken near Skukuza in the Kruger National Park (KNP), South Africa. Skukuza occurs at 365 m above sea level and has a mean annual rainfall of 550 mm, which typically falls between November and April. Mean annual minimum and maximum temperatures are 14.3 and 29.5 °C, respectively. Soils at the study site are derived from granites and are typically shallow (<1 m) with high sand content (>65%). The study site is mainly open woodland dominated by a mix of broad-leaved bush-willow (*Combretum*) and *Acacia* savanna types. The study site was located within the fence that encloses the Skukuza airport (−25.01016299S, 31.92083898E). The airport fence is well maintained and excludes all larger animals and includes a relatively large area (ca 50 ha) of natural vegetation that is burnt at a similar frequency to the surrounding landscape. The six selected species provide a reasonable sample of the functional diversity of trees in the study region and includes species that are known to have different drought responses (see Table 1).

### Diameter growth increment

A total of 54 trees (nine individuals for each of the six species) were fitted with manual band dendrometers (Agricultural Electronics Corporation, Tucson, AZ, USA). The dendrobands were installed during the first 2 weeks of October 2021. Measurements began in mid-November 2021. Changes in dendroband circumference were recorded every 2–3 weeks until the end of September 2023. These readings allowed us to calculate annual diameter increment (ADI) and peak diameter growth rate (PDGR), as well as the length of the growing season (November–April).

### Photosynthesis measurements

A LI-COR LI-6400XT infra-red gas analyser (IRGA; LI-COR Biosciences, Lincoln, NE, USA) portable photosynthesis system was used to measure light and CO_2_ response curves for the six study species during the peak of the growing season months of both the 2021/22 and 2022/23 rainfall years. Logistical constraints prevented us from measuring gas exchange in the field. That is, even though branch excision can alter physiological responses (Santiago and Mulkey 2003), we used excised branches. Branches (>1 m) were collected from trees growing at the study site. Branch ends were placed in water and then brought back to the laboratory in Skukuza for photosynthesis measurements. Leaves were clamped in the chamber, the chamber block temperature was set close to ambient laboratory temperature and the chamber humidity was regulated to be in the range 40–60% RH. The artificial chamber light source was set to 2000 𝜇mol m^−2^^−1^, and the chamber CO_2_ concentration was set to 400 p.p.m. Once steady-state photosynthesis has been achieved, a light response curve was generated by reducing the light in the following steps: 2000, 1500, 1000, 500, 250, 120, 60, 30, 15 and 0 𝜇mol m^−2^^−1^. For the CO_2_ response curves, the same setup and procedure were used. Once steady-state gas exchange was achieved, the chamber CO_2_ concentration was changed in the following sequence: 400, 300, 200, 100, 50, 400, 400, 400, 600, 800, 1000 and 1200 p.p.m. The switch from 50 to 400 p.p.m. can produce anomalous gas exchange estimates; visual inspection of the response curves was used to eliminate such anomalous data points prior to analysis. The cleaned response curves were used to estimate the maximum rate of carboxylation (${{Vc}}_{{max}}$), the rate of electron transport at 2000 𝜇mol m^−2^ s^−1^ (*J_max_*) and the maximum rate of photosynthesis (*A_max_*).

### Xylem pressure potentials

Once a month, we measured predawn and midday xylem pressure potentials using a Scholander-type pressure chamber (Model 1505D, PMS Instrument Company, Albany, OR, USA). Measurements were made on freshly clipped twigs with fully expanded healthy leaves from the 54 trees (nine individuals for each of the six species) at the Skukuza airport study site. Predawn (${{\psi}}_{{pd}}$) and midday measurements (${{\psi}}_{{md}}$) were taken every month during the 2021/22, 2022/23 and 2023/24 growing seasons. To measure soil water potentials at the study site, three TEROS-21 water potential sensors (Meter Group) were buried at three depths (15, 30 and 45 cm) in the centre of the study site. Soil water potentials were logged every 5 min using a ZL-6 data logger (Meter Group).

### Shoot drying curves

We estimated whole shoot saturated water content (${SWC}$_br_), relative water content at turgor loss point (${RWC}$_tlp_), relative water content at water potential at 50% loss of hydraulic conductivity (${RWC}$_50_) and standardized capacitance (*C*_std_) using shoot drying curves (see Figure S1 available as Supplementary Data at *Tree Physiology* Online). Shoot relative water content and corresponding xylem water potentials were measured on four sun-exposed branches collected on healthy adult trees growing at the study site. Branch lengths were at least 1.5 times the species’ maximum vessel length. Branches of 1–1.2 m, with basal diameters of 10–14 mm, were measured for each species. In order to ensure full saturation, branches were either sampled at the end of the afternoon and stored overnight in cool and humid opaque plastic bags or early in the morning immediately before the start of the measurements.

Shoots’ proximal ends were cut off several times under water to relax the xylem tension and then were sealed with parafilm at the start of the measurements. We followed the methods described by Waite et al. (2024), except that we associated pneumatic measurements to estimate branch vulnerability to drought-induced embolisms. We weighed each shoot at increasing drying steps at 10 mg precision to quantify water loss. At each drying step, the water potential of two leaves per shoot was measured using a Scholander pressure chamber (PMS Instruments). Shoots were dried down until constant mass or the maximum water potential measurable by the pressure chamber (−10 MPa) was reached. The initial mass at the beginning of the experiment corresponds to the water-saturated mass (SM) and was always above −0.3 MPa. All subsequent fresh mass (FM) measurements were performed in a series of drying steps with increasing drying intervals of up to 4 h and usually included more than five measurements depending on species identity and measurement conditions. For each drying step, before measuring mass and water potential, shoots were placed in opaque plastic bags holding humidified towels for 30–60 min to limit transpiration and equilibrate the water potential (Rodriguez-Dominguez et al. 2022). In addition, the mass of the excised leaves at each drying step was documented before water potential measurements and all cut-off and shed leaves were weighed and stored. Subsequently, the samples were oven-dried at 75 °C for 72 h to estimate their dry mass (DM). Based on the shoot fresh mass at each drying step, the mass of the removed leaves and the corresponding DM, we computed shoot relative water content for each drying step *i* > 1 (at the first step, RWC is equal to 1 by definition):


(1) \begin{equation*} {RWC}_i=\frac{SM\ast{\varPi}_1^i\frac{FM_i}{FM_{i-1}}- DM}{SM- DM}\quad (\textrm{g}\ \textrm{g}^{-1}) \end{equation*}

where ${{FM}}_{{i}}$ refers to the fresh mass after each drying step before leaf cutting, and ${{FM}}_{{i}-1}$ refers to the fresh mass of the previous drying step after leaves were cut off. We estimated the whole shoot saturated water content (${SWC}$_branch_) as $\frac{SM- DM}{DM}.\kern0.5em$To estimate RWC at a given water potential for each species (*RWC*_tlp_ and *RWC*_50_), the relationship between RWC and water potential was estimated with an exponential decay equation. The exponential decay function described the relationship better than a linear decay function (see methods in Li et al. 2018, Blackman et al. 2019 and Waite et al. 2024). To estimate species’ capacitance values, we first fitted a linear regression on the linear portion of the relationship between RWC and water potential after stomatal closure (see Figure S1 available as Supplementary Data at *Tree Physiology* Online). We used the slope of the relationship to compare species. The slope was then standardized to account for shoot DM:


(2) \begin{equation*} C=\frac{\Delta RWC}{\Delta \varPsi}\ast \frac{SM- DM}{DM}\ast \frac{1}{Mw}\quad (\textrm{mol}\ \textrm{kg}^{-1}\ \textrm{MPa}^{-1}) \end{equation*}

where $\frac{\Delta RWC}{\Delta \psi }$ is the slope of the relationship between RWC and water potential (g g^−1^ MPa^−1^), multiplied by the maximum amount of stored water by the shoot (SM – DM, g) and divided by shoot DM (kg). The resulting value (g kg^−1^ MPa^−1^) was then converted to mol kg^−1^ MPa^−1^ by dividing it by the molar mass of water (Mw; 18.01 g mol^−1^).

### Vulnerability curves

We measured branch xylem vulnerability curves to estimate the water potential at 50% loss of hydraulic conductivity (${P}$_50_) on a subset of the branches used for drying curve measurements (*n* = 3– 5 per species, see Figure S2 available as Supplementary Data at *Tree Physiology* Online) using the pneumatic method (Pereira et al. 2016, Paligi et al. 2023). We used a Pneumatron device alongside the drying curve measurements (i.e., in addition to weighing and measuring water potential of each shoot) (Pereira et al. 2020) at each of the drying steps. The reservoir pressure was tracked with the Pneumatron in 0.5-s intervals over 1.5 min per measurement (including a pump time of ~2 s in semi-automated mode). The amount of air discharged (AD) into the reservoir (i.e., tubing and branch xylem) was calculated based on the ideal gas law following Pereira et al. (2016). The maximum detectable amount of AD is associated with a change of pressure in the system by ca 50 kPa (Pereira et al. 2020). We prepared the branches as described in the previous section, except that we sealed potential leakage points (e.g., scars, cut leaves) using a fast-drying contact adhesive (Loctite 431 with activator SF 7452; Henkel, Düsseldorf, Germany) to minimize air entry from outside and isolate the branches’ xylem. We also cut the basal ends of the branches with a sharp razor blade to clear obstructions for airflow (Pereira et al. 2016, Jansen et al. 2019). At each drying step (cf. shoot drying curves), a branch was connected to the Pneumatron using rigid tubing tightened with hose clamps. The volume of the elastic tube was kept as small as possible (1.7–3 mL) to minimize pressure-dependent changes in reservoir volume (Pereira et al. 2020, Paligi et al. 2023). We then followed the same protocol as for the drying curves, increasing drying intervals to induce embolism (Sperry et al. 1988) as well as measuring AD and water potential on two leaves after an equilibration step. As per the drying curves, we simultaneously measured multiple branches. The AD measurements were taken until constancy in AD in consecutive measurements or until a xylem water potential of −10 MPa was reached. This resulted in measurement durations of 3–7 days, as well as in 10–20 AD and leaf water potential measurements per branch. The percentage of AD (PAD) was calculated as described by Paligi et al. (2023):


(3) \begin{equation*} {PAD}_i=100 \ast \frac{AD_i-{AD}_{min}}{AD_{max}-{AD}_{min}}(\%) \end{equation*}

where ${{AD}}_{{i}}$ is the amount of air discharged for measurement ${i}$, ${AD}$_min_ is the minimum amount of air discharged from the fully hydrated branch and ${AD}$_max_ is the maximum amount of air discharged from the branch when completely desiccated.

### Leaf traits

On additional healthy branches, we measured the water potential at leaf turgor loss point (${\psi}$_tlp_), SLA and leaf cuticular conductance (${g}$_min_) on fully expanded sun-exposed leaves. ${\psi}$_tlp_ was estimated using two leaves per tree (${n}$ = 4 individuals per species) following Bartlett et al. (2012). The leaves were cut off, cleaned, double-bagged with humidified towels and stored at 4 *°*C until measurement. Subsequently, leaf discs were cut out, covered with aluminium foil and submerged in liquid nitrogen for at least 2 min before measuring osmolality (mmol kg^*−*1^) at full turgor with a vapour pressure osmometer (VAPRO 5600, Wescor, USA), which was then used to estimate ${\psi}$_tlp_ using the equation in Bartlett et al. (2012). SLA (cm^2^ g^*−*1^) was measured on 8–50 leaves per tree from healthy individuals (${n}$ = 4 per species). To compute SLA, we divided the leaf surface area (cm^2^) computed from scans using ImageJ by their oven-dried mass (g). ${g}$_min_ was measured on two to three mature, healthy leaves per tree (${n}$ = 4 per species). These leaves were initially fully hydrated overnight. We then excised the leaves, recorded their saturated leaf mass at 0.1 mg precision, sealed the cut with wax and scanned them to calculate their area using ImageJ. Leaves were desiccated on a metal grid in a temperature-controlled chamber for 5–6 h while documenting temperature and relative humidity. Leaf mass was recorded at 30- to 45-min intervals. Subsequently, samples were oven-dried at 70 *°*C for 72 h, and their DM was measured. The projected leaf area was multiplied by two to account for the total leaf surface. The transpiration rate ${J}$(mmol m^*−*2^ s^*−*1^) was approximated as the slope of the linear regression between leaf mass and time (g s^*−*1^; see Sack and Scoffoni (2011), divided by the leaf area (m^2^) and the millimolar mass of water (1$.$8015 *×* 10^*−*2^ g mmol^*−*1^). Cuticular conductance ${g}$_min_ (mmol m^*−*2^ s^*−*1^) was then calculated as ${g}$_min_ = ${J}\times{P}$_amb_$/$VPD, where ${P}$_amb_ is the ambient pressure (kPa), VPD is the vapour pressure deficit (kPa) and their ratio VPD/${P}$_amb_ is the difference in mole fraction of water vapour inside and outside the leaf as the driving force of diffusion over the leaf surface, assuming water vapour saturation in the mesophyll (cf. Nobel (2020), pp 379).

### Wood traits

We sampled a wood segment on each branch to determine stem WD (g cm^*−*3^). We measured the saturated mass of the debarked samples and then the fresh volume using the water displacement method (Osazuwa-Peters and Zanne 2011) and the oven-dried mass (105 °C for 72 h). WD was estimated as the ratio between DM and fresh volume.

### Statistical analyses

We estimated growth parameters using Bayesian Linear Mixed-Effects models (LMEs). The stem growth analyses used hierarchical Bayesian analyses that considered stem size and the observation year as random effects. The photosynthesis analyses used hierarchical Bayesian analyses of steady-state gas exchange measurements and light and CO_2_ response curves using methods developed by (Patrick et al. 2009). We assessed associations between traits based on a Pearson correlation matrix plotted with R package corrmorant v0.0.0.9007 (Link 2020). A species by trait matrix was analysed using principal components analysis (PCA) using base R. The PCA was plotted using ggbiplot.

## Results

### Species-level variation in growth and photosynthetic traits

**Figure 1:**
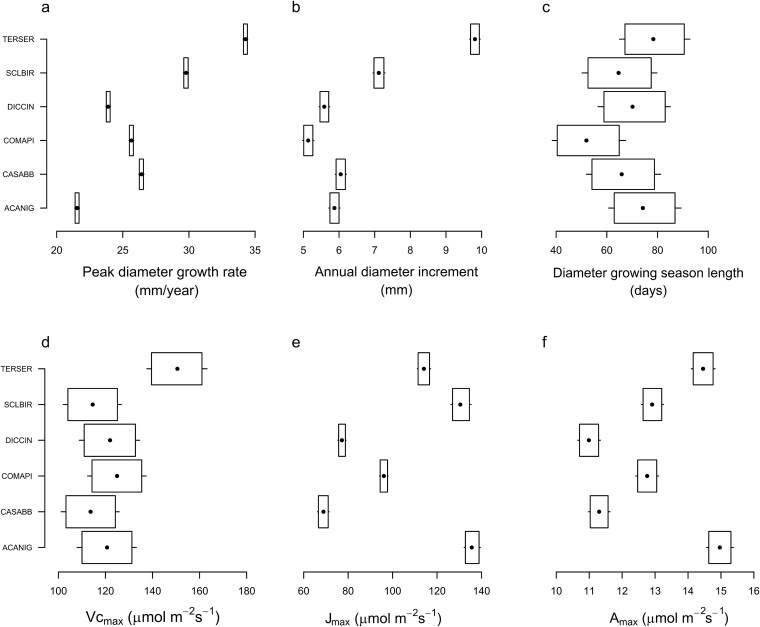
Stem growth parameters and photosynthesis measurements of the study species. The data analysed in (a–c) included the dendrometer band data from the 2021/22 and 2022/23 rainfall years. Panels (d–f) show leaf-level gas exchange parameters of the study species. For all panels, the points represent the posterior means, the boxes the 90% credible intervals and the whiskers the 95% credible intervals of the parameter estimates. Species labels are abbreviations of the full names as shown in Table 1.

Growth rates and timing of growth differed greatly among the six species. *Terminalia sericea* and *Sclerocarya birrea* consistently had the highest PDGR, 34.3 (34.1, 34.4) and 29.8 (29.6, 30.0) mm year^*−*1^ respectively, where values in parentheses indicate the 95% credible intervals. *Terminalia sericea* and *S. birrea* also had the highest ADI, 9.83 (9.65, 9.98) and 7.14 (6.94, 7.30) mm, as well as the longest growing season among all species (Figure 1a–c). *Combretum apiculatum* and *Dichrostachys cinerea* were among the slowest growing (ADI = 5.15 and 5.60 mm, respectively, as well as PDGR = 25.67 and 26.42 mm year^*−*1^, respectively). *Acacia nigrescens* had the lowest peak diameter growth rate of 21.56 (21.41, 21.73) mm year^*−*1^ and an intermediate ADI 5.89 (5.70, 6.04) mm compared with the other species (Figure 1a–c).

We observed clear differences among species leaf-level gas exchange parameters; *T. sericea* had the highest *Vc_max_* 150.34 (136.62, 163.53) *μ*mol m^*−*2^ s^*−*1^. For the other five species, *Vc_max_* was within the same range (113.69*–*121.96) *μ*mol m^*−*2^ s^*−*1^ (Figure 1d–f). *Terminalia sericea* and *S. birrea* also both had among the highest *J_max_* 114.52 (11.56, 116.99) and 130.78 (125.70, 135.45) *μ*mol m^*−*2^ s^*−*1^, respectively and among the highest assimilation *A_max_* 14.45 (14.10, 14.82) and 12.90 (12.56, 13.24) *μ*mol m^*−*2^ s^*−*1^, respectively. Despite not having a high growth rate, *Acacia nigrescens* had the highest *J_max_* 135.58 (131.42, 139.70) *μ*mol m^*−*2^ s^*−*1^) and the highest *A_max_* 14.96 (14.55, 15.37) *μ*mol m^*−*2^ s^*−*1^ (Figure 1d–f).

### Species hydraulic parameters

**Figure 2:**
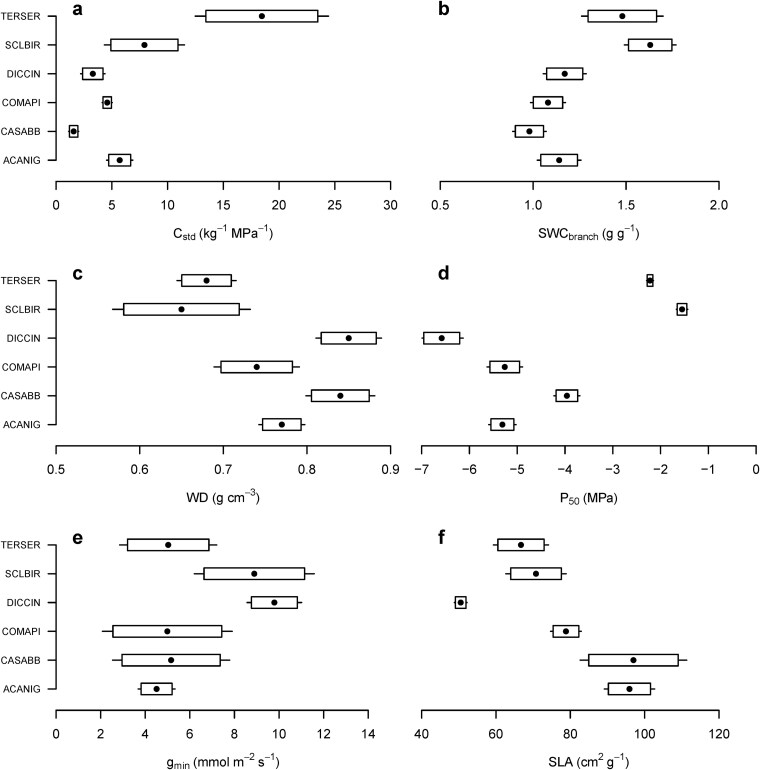
Mean *±* 95% CIs for six traits including: water storage traits, (a) shoot hydraulic capacitance (${C}$_std_) and (b) saturated water content of the shoot (SWC_branch_); xylem safety traits, (c) branch wood density (WD) and (d) water potential at 50% loss of hydraulic conductivity (${P}$_50_); (e) the residual conductance trait, leaf cuticular conductance (${g}$_min_); and (f) specific leaf area (SLA). For all panels, points represent the means, boxes the 90% confidence intervals and whiskers the 95% confidence intervals of each trait. Species labels are as in Table 1.

Whole shoot hydraulic capacitance (*C_std_*) and saturated water content *SWC_branch_* were higher in *S. birrea* and *T. sericea* compared with the other four species. *Cassia abbreviata* had the lowest *C_std_* and *SWC*_branch_ values (Figure 2a and b, Table 3). The relative water content at turgor loss point (*RWC_tlp_*), an indicator of the water stored in branches at wilting point, was lowest in *D. cinerea* but similar among the other species (Table 3). *Sclerocarya birrea* had the least negative *P*_50_ (*−*1.5 *±* 0.07 MPa), followed by *T. sericea* (*−*2.2 *±* 0.24 MPa). In contrast, *D. cinerea* had a markedly more negative *P*_50_ (*−*6.6 *±* 1.0 MPa), while the other three species ranged between −4 and − 5.3 MPa (Figure 2d). Wood density (WD) was highest in *D. cinerea* and *Cassia abbreviata* (ca 0.84 cm^3^ g^*−*1^), whereas *S. birrea* and *T. sericea* had the lowest WD (ca 0.66 cm^3^ g^*−*1^, Figure 2c). Likewise, *ψ_tlp_* was most negative for *D. cinerea* (*−*2.99 *±* 0.2 MPa; Table 3) and most positive for *T. sericea* at approximately half that water potential (1.40 *±* 0.04 MPa). *ψ*_tlp_ values for the remaining species ranged between −1.8 and −2 MPa (Table 3). Interestingly, we found that *D. cinerea* had the highest leaf residual conductance (${{g}}_{{min}}$; 9.8 *±* 0.62 mmol m^*−*2^ s^*−*1^) followed by *S. birrea*, which showed high variability (8.9 *±* 1.37 mmol m^*−*2^ s^*−*1^). The remaining four species had mean values ranging between 4.5 and 5.2 mmol m^*−*2^ s^*−*1^ (Figure 2e). Mean SLA values of the six species ranged between 50.5 cm^2^ g^*−*1^ in *D. cinerea* and between 96 and 97 cm^2^ g^*−*1^ in *A. nigrescens* and *Cassia abbreviata*. The remaining three species had mean SLA values in the range of 67–79 cm^2^ g^*−*1^ (Figure 2f, Table 3).

**Table 3:** Mean values and 95% confidence interval for the trait estimates for each of the study species

Species	Trait mean *±* 95% CI			
	${\boldsymbol{P}}$ _50_	WD	${{\boldsymbol{\psi}}}_{{\boldsymbol{tlp}}}$	SLA
*Acacia nigrescens*	*−*5$.$31 *±* 0$.$32	0$.$77 *±* 0$.$03	*−*1$.$85 *±* 0$.$09	95$.$90 *±* 6$.$70
*Cassia abbreviata*	*−*3$.$96 *±* 0$.$79	0$.$84 *±* 0$.$04	*−*2$.$05 *±* 0$.$44	97$.$0 *±* 14$.$2
*Combretum apiculatum*	*−*5$.$26 *±* 0$.$75	0$.$74 *±* 0$.$05	*−*1$.$79 *±* 0$.$05	78$.$80 *±* 4$.$09
*Dichrostachys cinerea*	*−*6$.$58 *±* 1$.$96	0$.$85 *±* 0$.$04	*−*2$.$99 *±* 0$.$49	50$.$50 *±* 1$.$69
*Sclerocarya birrea*	*−*1$.$55 *±* 0$.$14	0$.$65 *±* 0$.$08	*−*2$.$02 *±* 0$.$15	70$.$80 *±* 8$.$05
*Terminalia sericea*	*−*2$.$22 *±* 0$.$46	0$.$68 *±* 0$.$04	*−*1$.$40 *±* 0$.$10	66$.$70 *±* 7$.$35
	** $\boldsymbol{C_{std}}$ **	** ${{\boldsymbol{SWC}}}_{{\boldsymbol{br}}}$ **	** ${\boldsymbol{{RWC}}}_{\boldsymbol{{tlp}}}$ **	***^g^* $_{\boldsymbol{min}}$ **
*Acacia nigrescens*	5$.$71 *±* 1$.$16	1$.$14 *±* 0$.$12	0$.$94 *±* 0$.$05	4$.$51 *±* 0$.$82
*Cassia abbreviata*	1$.$58 *±* 0$.$44	0$.$98 *±* 0$.$09	0$.$85 *±* 0$.$03	5$.$16 *±* 2$.$60
*Combretum apiculatum*	4$.$59 *±* 0$.$45	1$.$08 *±* 0$.$09	0$.$87 *±* 0$.$02	4$.$99 *±* 2$.$88
*Dichrostachys cinerea*	3$.$30 *±* 1$.$08	1$.$17 *±* 0$.$11	0$.$71 *±* 0$.$05	9$.$79 *±* 1$.$21
*Sclerocarya birrea*	7$.$93 *±* 3$.$56	1$.$63 *±* 0$.$14	0$.$86 *±* 0$.$05	8$.$89 *±* 2$.$66
*Terminalia sericea*	18$.$46 *±* 5$.$93	1$.$48 *±* 0$.$22	0$.$90 *±* 0$.$03	5$.$03 *±* 2$.$16

**Figure 3:**
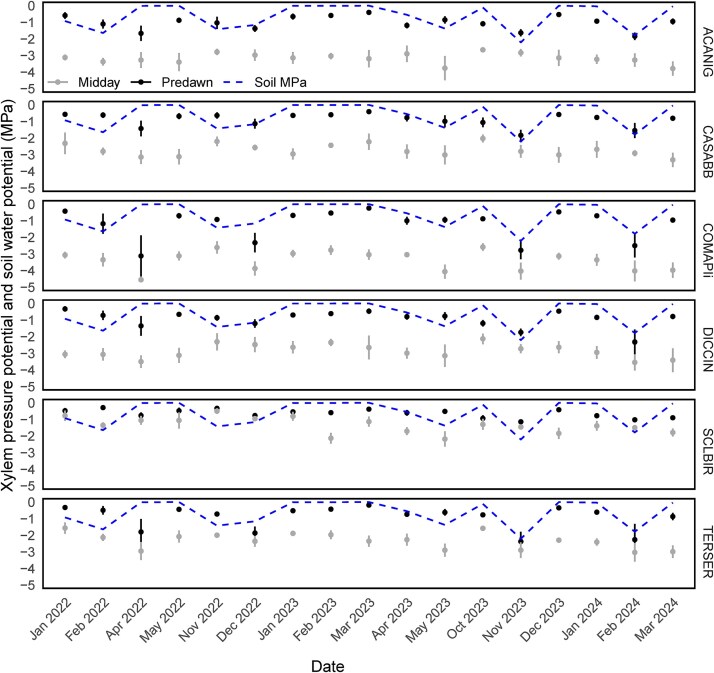
Xylem pressure potentials of the study species. Mean *±* 95% confidence intervals for predawn and midday xylem pressure potentials and soil water potentials (average of 15, 30 and 45 cm depths) for the growing season months between January 2022 and April 2024. Predawn and midday XPPs were measured on nine replicates for each of the six species (54 trees).

*Sclerocarya birrea* consistently had the least negative *ψ_pd_*, even during times of low soil water availability and the smallest difference between *ψ_md_* and *ψ_pd_*. *ψ_pd_* in *T. sericea* tended to become more negative (or decrease) during times of low soil water availability, while the difference between *ψ_md_* and *ψ_pd_* tended to be small. *ψ_pd_* during times of low soil water availability were most negative in *C. apiculatum*. *Acacia nigrescens* and *C. apiculatum* had the biggest differences between *ψ_md_* and *ψ_pd_* (Figure 3).

### Relationships among traits

**Figure 4:**
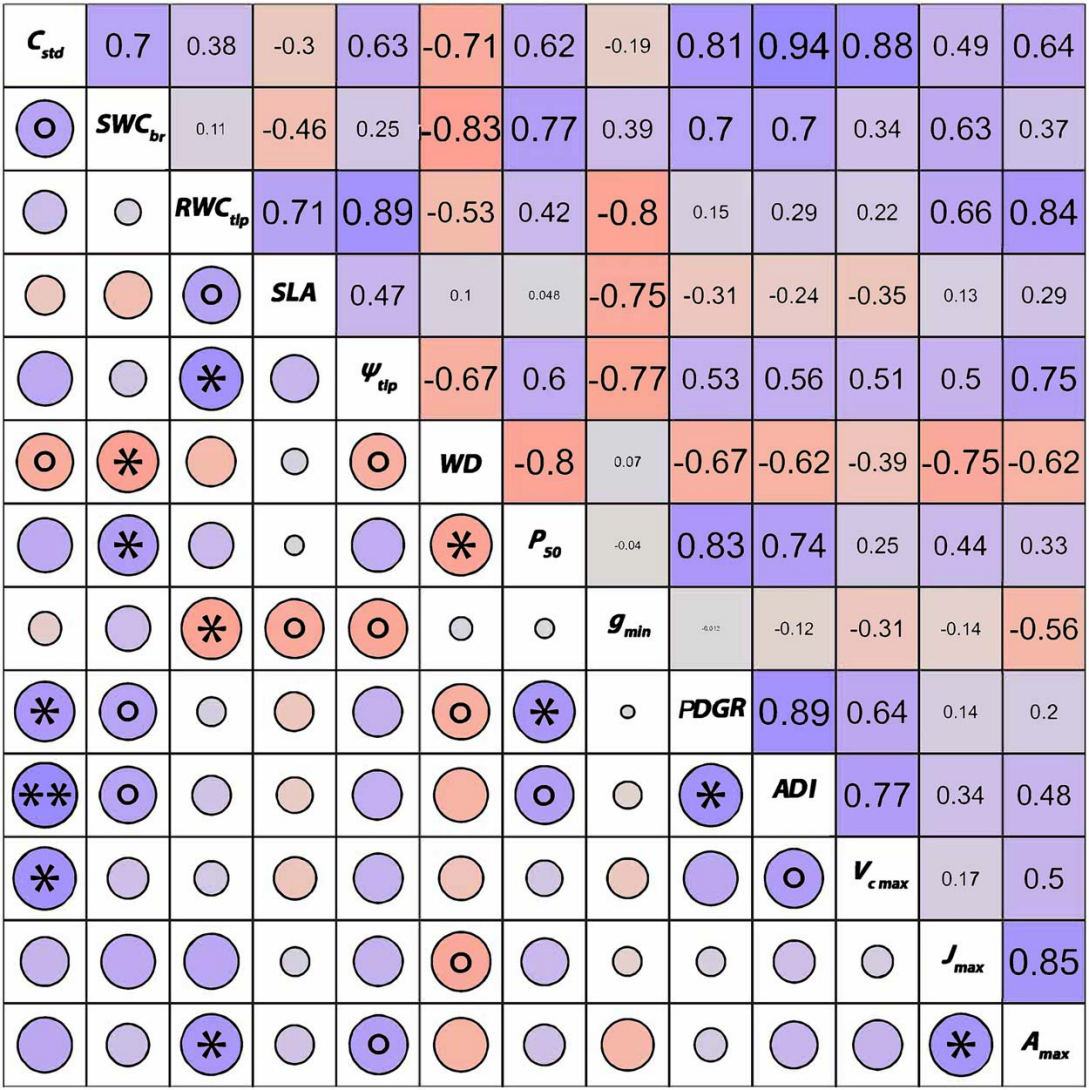
Correlation matrix of investigated traits in the six species. Presented here are standardized shoot hydraulic capacitance (*C*_std_), saturated water content of the shoot (${SWC}$_br_), relative water content at turgor loss point (${RWC}$_tlp_), specific leaf area (SLA), water potential at leaf turgor loss point (−${\psi}$_tlp_), branch wood density (WD), water potential at 50% loss of hydraulic conductivity (*P*_50_), leaf residual conductance (${{g}}_{{min}}$), peak diameter growth rate (PDGR), annual diameter increment (ADI), maximum carboxylation rate of Rubisco (${V}{{c}}_{{max}}$), rate of electron transport at 2000 ${\mu}$mol m^2^ s^*−*1^ photon flux density (${{J}}_{{max}}$) and maximum rate of photosynthesis (${{A}}_{{max}}$). Units and definitions can be found in Table 2. Asterisks and circles indicate significant (^*^ = *P* < .05, and ^**^ = *P* < .01) and marginally significant (*P* < 0.10) relationships, respectively.

We detected significant associations among measured traits. As expected, we found that across all species, ADI and PDGR were positively correlated (*r* = 0.89, *P* = 0.02, Figure 4 and see Supplementary Table S1 available as Supplementary Data at *Tree Physiology* Online). *V_cmax_* was the photosynthetic parameter most strongly related to growth patterns in the six species (e.g., correlation with ADI, *r* = 0.77, *P* = 0.07). By contrast, *J_max_* and *A_max_* were poorly associated with the growth parameters (Figure 2). Strong correlations were observed between *C_std_* and *SWC_branch_* (*r* = 0.70), as well as between both *C_std_* and *SWC_br_* with WD (*r* = −0.71 and − 0.83, respectively) and *P*_50_ (*r* = 0.63 and 0.77, respectively). Additionally, *C_std_* was positively correlated with *ψ_tlp_* (*r* = 0.63). These findings suggest that the study species with high water storage and hydraulic capacitance tend to have lower WD, xylem that is less resistant to embolisms and leaves that are less resistant to wilting. Moreover, *RWC_tlp_* was strongly associated with SLA (*r* = 0.71) and ${{g}}_{{min}}$ (*r* = −0.80), indicating that species with high SLA and with a lower residual conductance tend to store more water at the leaf wilting point compared with other species.

Furthermore, we identified an inverse relationship between *P*_50_ and WD (*r* = −0.80), indicating that species with higher WD tend to have xylem that is more resistant to embolisms.

*C_std_* and *SWC_br_* were strongly and positively correlated with PDGR (*r* = 0.81 and 0.70, respectively) and ADI (*r* = 0.94 and 0.70, respectively). *C_std_* was also associated with the maximum rate of net photosynthesis (*A_max_*; *r* = 0.64) while *SWC_branch_* was related to the electron transport at high light intensity (*J_max_*; *r* = 0.63). Thus, study species with higher hydraulic storage and capacitance tended to grow faster and have higher photosynthetic rates compared with the other species. Similarly, we observed that *RWC*_tlp_ was related to photosynthetic parameters, specifically with *A_m_* (*r* = 0.66) and *J_max_* (*r* = 0.84). This suggests that species with higher water storage at the turgor loss point also had higher photosynthetic rates, indicating a possible tendency towards conservative water use while maintaining high photosynthetic performance.

We found that WD and *P*_50_ were also related to the growth performance indicators. Specifically, WD exhibited negative correlations with PDGR (*r* = −0.67) and with ADI (*r* = −0.62), while *P*_50_ showed positive correlations with PDGR (*r* = 0.83) and ADI (*r* = 0.74). These findings suggest that species with lower xylem vulnerability grow more slowly.

Nonetheless, only WD and *P*_50_ were related to photosynthetic rates of the species (*r* = −0.75 and *r* = −0.62 with *J_max_* and *A_m_*, respectively). *ψ_tlp_* was also related to *A_max_* (*r* = 0.85), indicating that species with leaves more vulnerable to drought can achieve higher CO_2_ assimilation rates.

**Figure 5:**
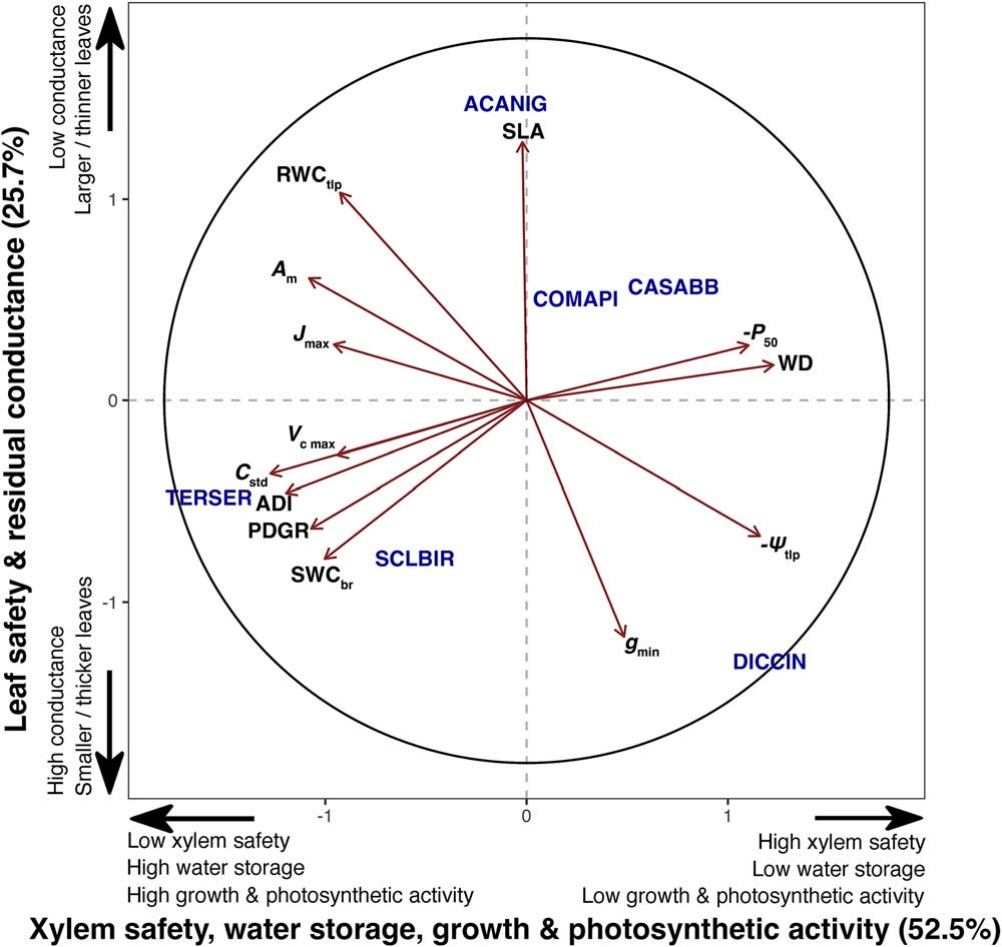
Principal component analysis (PCA) showing the variation in growth, leaf-level gas exchange parameters, xylem and leaf safety–related traits among the six study species. Presented here are (i) growth-related traits; peak diameter growth rate (PDGR), annual diameter increment (ADI). (ii) Leaf-level gas exchange parameters; ${V}\ {{c}}_{{max}}$, ${{J}}_{{max}}$ and ${{A}}_{{max}}$. (iii) Xylem safety traits; inverse water potential at 50% loss of hydraulic conductivity (-*P*_50_) and branch wood density (WD). (iv) Leaf safety trait; water potential at leaf turgor loss point (-*P_tlp_*). (v) Water storage traits; standardized shoot hydraulic capacitance (*C_std_*), saturated water content of the shoot (SWC_br_), and relative water content at turgor loss point (RWC*_tlp_*). (vi) Leaf ecological trait; specific leaf area (SLA). (vii) Residual conductance traits: leaf cuticular conductance (${{g}}_{{min}}$). Species names are abbreviations of the full names provided in Table 1.

We used the traits shown in Figure 2 to conduct a principal components analysis (Figure 5). The first principal component axis of the PCA explained 52% of the variance in the trait data and separated species with high values for photosynthesis, growth and water storage–related traits from species characterized by leaf and xylem parameters associated with drought tolerance (see Table S2 available as Supplementary Data at *Tree Physiology* Online). For example, *T. sericea* had the highest *V c_max_* values, the highest growth rates (Figure 1) and some of the highest water storage parameters (Figure 4) while *D. cinerea* was at the opposite extent of PC1 with the highest WD, leaf and xylem safety parameters (*P*_50_ and *ψ*_50_). The second principal component axis explained an additional 23% of the variance and separated species with high SLA and *RWC_tlp_* from species with high ${{g}}_{{min}}$. *Acacia nigrescens* had the highest values of SLA and *RWC_tlp_*, and *D. cinerea* had the highest ${{g}}_{{min}}$ (see Table S3 available as Supplementary Data at *Tree Physiology* Online).

## Discussion

Here, we set out to determine if there is a relationship between drought tolerance and general performance under optimal conditions (growth/photosynthetic rates) in a semi-arid savanna woody community. We specifically asked whether there is a trade-off where savanna trees that are more drought-tolerant exhibit slower growth and lower photosynthetic rates, while those with higher photosynthetic and growth rates show less tolerance to drought (drought avoiders), reflecting distinct hydraulic and growth strategies. Rather than discrete and mutually exclusive strategies, our findings support two main trait syndromes, reflecting trade-offs between hydraulic and growth strategies, arrayed along a continuum with species presenting intermediate trait values.

### Species differences along a growth–drought tolerance continuum

We found substantial differences in peak growth rates among the six species with a difference of >14 mm diameter growth per year between the slowest growing (*A. nigrescens*) and fastest growing species (*T. sericea*). Big differences were also evident (almost double) in annual diameter increments, with the lowest annual increment in *C. apiculatum* and the highest annual growth in *T. sericea*. The faster-growing species (*T. sericea* and *S. birrea*) also tended to have higher photosynthetic efficiencies (i.e., *V_Cmax_*, *J_max_*, *A_max_*).

Following Wright et al. (2004), a positive relationship between SLA and ${{g}}_{{min}}$ was expected as plants with higher SLA are expected to have higher leaf conductance rates and thus higher residual conductance enhanced by an increased surface area, to maximize carbon gain during favourable conditions, while species with lower SLA are expected to be more conservative in their water use. Here, we interestingly observed a negative SLA–${{g}}_{{min}}$ relationship. Across woody datasets, ${{g}}_{{min}}$ often declines with increasing LMA (i.e., lower SLA), implying that species with higher SLA typically have higher ${{g}}_{{min}}$ (Slot et al. 2021). However, past studies emphasize that ${{g}}_{{min}}$ is multi-factorial (cuticle chemistry, structure and temperature) and not reliably predicted by SLA or thickness alone, so interspecific correlations can be weak or context-dependent (Duursma et al. 2019). Thus, our inverse pattern may reflect savanna-specific trait combinations where species with larger and thinner leaves have to compensate by investing in tighter stomatal sealing efficiency or an elaborate wax chemistry (see Wang et al. 2024). In our study, *D. cinerea* had the lowest SLA and also the highest ${{g}}_{{min}}$ while *Acacia nigrescens* had the highest SLA and the lowest ${{g}}_{{min}}$. Given its trait combination (low SLA with high ${{g}}_{{min}}$), *D. cinerea* would be expected to face greater vulnerability to drought as water deficits intensify, unless offset by other whole-plant adjustments. However, *D. cinerea* also exhibited the most negative *P*_50_ (i.e., the highest resistance to cavitation), indicating substantial xylem safety despite elevated ${{g}}_{{min}}$. Taken together with its low capacitance, this suggests that *D. cinerea* may be less prone to embolism per se but could experience shorter time-to-hydraulic-failure under prolonged deficits due to residual water loss.

Another species with an extreme strategy, albeit at the other end of the trait spectrum, is *S. birrea*. High capacitance values confirmed field observations of these large trees, which consistently possess water-rich wood and thick, spongy bark. This species seemed to rely mostly on water storage to cope with seasonal drought. Interestingly, we recorded midday water potentials in *S. birrea* that were more negative than its stem *P*_50_, yet we did not observe any visible damage or dieback in those individuals. One explanation could be that the species presents a high degree of hydraulic/vulnerability segmentation where distal organs are more vulnerable to cavitation and can shed or fail first, which relieves tension and protects the more vital, carbon-costly organs (e.g., main stem) from catastrophic hydraulic failure (Song et al. 2022). In essence, *S. birrea* may sacrifice ‘expendable’ tissues (through early embolism and leaf drop) to safeguard its trunk xylem integrity during severe drought. Nonetheless, it is important to note that measurement artefacts could have contributed to the apparent discrepancy between *P*_50_ and observed water potentials. The exceptionally high water content in tissues outside the xylem (e.g., living bark and parenchyma) may introduce noise in pressure chamber readings.

When considering the overall trait coordination space, both *S. birrea* and *D. cinerea* appear to have two extremely different strategies (i.e., low xylem resistance/high-capacitance end of the continuum for the first one and high xylem resistance/low-capacitance end for the second one). *Dichrostachys cinerea* had very high resistance to cavitation, high WD, low SLA and high ${{g}}_{{min}}$ but the lowest water storage capacity. Coupled to that, *D. cinerea* was one of the slowest-growing species and had the lowest peak photosynthetic rate. In that case, there is a clear trade-off between performance under optimal conditions and drought tolerance traits. In contrast, *S. birrea*, together with *T. sericea*, seems to occupy the opposite end of the trait spectrum with low resistance to cavitation and the lowest wood densities but a high capacity for water storage. These findings are in agreement with previous studies done on karst tree species that found an inverse relationship between water storage capacity and xylem embolism resistance (Liu et al. 2024). *Sclerocarya birrea* and *T. sericea* also had the smallest differences between their predawn and midday xylem pressure potentials, suggesting that they either had access to deeper soil water or could be utilizing stored water (high *C*_std_) during times of limited soil water availability. The remaining species were intermediate on the xylem safety–capacitance–growth spectrum and showed lower conductance and higher SLA than the fast-growing pair (*T. sericea* and *S. birrea*).

### Short vs prolonged drought: ecological implications of contrasting strategies

We have shown that in these semi-arid South African savannas, co-occurring deciduous tree species present a continuum of hydraulic strategies with both ends of the trait spectrum represented by extreme strategies with clear trade-offs between drought tolerance and performance under optimal conditions. However, it is not clear what the consequences of this large spectrum and trade-off will be for shorter-growing season dry spells, as well as more extensive multi-season droughts. Wigley et al. (2024) investigated how the growth of the six study species was affected by growing season dry spells (i.e., short dry spells) and found that both *T. sericea* and *S. birrea* showed the largest decreases (i.e., shrinkage) in stem diameter during a growing season dry spell (likely due to high capacitance) but the highest growth rates during times of high soil water availability compared with other species. *Dichrostachys cinerea*, on the other hand, stopped growing but did not shrink during the growing season dry spell and had the lowest growth during times of high water availability (see Figure S3 available as Supplementary Data at *Tree Physiology* Online). This seems to confirm that for *T. sericea* and *S. birrea*, water storage acts as a buffer against seasonal drought variations, allowing them to go through short dry spells and start growing again as soon as soil water has been replenished. In contrast to this, species like *D. cinerea* seem to rely more on a resistant xylem, which might be more adequate for prolonged multi-seasonal droughts. Our results suggest that species with higher hydraulic capacitance are more adapted to survive short dry periods and are able to maximize performance during short rainfall periods, but their low resistance to cavitation could make them more vulnerable to hydraulic failure during extended drought conditions.

### Linking trait coordination to observed drought mortality

How do the short-term responses to low water availability translate to longer-term responses to droughts? Case et al. (2019, 2020) documented the response of three of the study species, *D. cinerea*, *T. sericea* and *S. birrea*, to an extensive drought that occurred between 2014 and 2016 in our study region. Two species with highly contrasting hydraulic strategies, *T. sericea* and *D. cinerea*, had the highest mortality rates, while *S. birrea* showed no drought-induced mortality. Our trait analyses suggest that *T. sericea* and *S. birrea* are drought-avoiding species that are able to survive shorter or growing season drought by using stored water reserves and are quickly able to resume high growth and photosynthetic rates once soil water becomes available again. The contrasting performance of the two species during the same drought suggests that unaccounted trait differences can confer a decisive advantage under dry conditions. A key factor may be rooting depth as it can be determinant to species survival in dry environments (Strohbach and Kutuahuripa 2014). *Sclerocarya birrea* is a large tree with a substantial root system (including a taproot penetrating ~2.4 m deep) that allows it to tap into deeper moisture reserves (Shone 1979, Hall et al. 2002), whereas *T. sericea* often has a shallow root system (<1 m deep in arid conditions; Strohbach and Kutuahuripa 2014). Another likely explanation is growth form and space occupation strategy. *Terminalia sericea* is smaller and resprouts readily after disturbances (e.g., fire), sending up multiple stems that quickly reclaim space (Hipondoka and Versfeld 2006), so the loss of any one stem has minimal impact on its overall population fitness. In contrast, *S. birrea* individuals are larger and more widely spaced (often solitary in open woodlands), reflecting a different strategy of niche occupation.

## Conclusion

Our findings show that co-occurring savanna tree species organize along a continuum with two end-member trait syndromes and intermediate/plastic strategies. The variation in functional traits among species suggests the presence of divergent trait syndromes. (i) Drought-avoiding species that are fast growing have a high capacitance and a low safety that buffer short deficits and quickly capitalize on favourable moisture. (ii) Drought-resistant species that are slow-growing have a high xylem safety and a low capacitance that withstand acute tension, which can be beneficial if drought persists. (iii) Intermediate species expressing context-dependent stomatal regulation with intermediate growth/photosynthesis and xylem safety. These contrasting syndromes explain short-term resilience to seasonal dry spells (e.g., Wigley et al. 2024). However, widespread mortality is more likely during extreme drought events for certain species or strategies that are more vulnerable to prolonged water stress (Case et al. 2020, Swemmer 2020). Deep-rooted species, such as *S. birrea* (Zhou et al. 2020), represent a notable exception, most likely due to their ability to access deeper soil water, which allows them to better withstand prolonged drought conditions (Case et al. 2020). These differential responses to drought ultimately influence species’ distribution patterns at the landscape scale (Case et al. 2020). The findings of Skelton et al. (2025), who compared the drought response of six evergreen species from South Africa’s Albany Subtropical Thicket, similarly show that co-occurring woody species adopt coordinated hydraulic trait syndromes reflecting trade-offs between safety, water storage and carbon gain. Together, both studies indicate that drought responses are best explained by integrated trait combinations and embolism dynamics rather than single traits, reinforcing the trait-based framework presented here.

## Supplementary Material

Supplementary_materials_tpag024

## Data Availability

The data underlying this article will be shared on reasonable request to the corresponding author.
