# Supplementary material for: Linking hydraulic strategy, drought response and carbon gain in co-existing savanna tree species
Source: Tree Physiol. 2026 Feb 20;46(3):tpag024. doi: 10.1093/treephys/tpag024 (PMC13037480; doi:10.1093/treephys/tpag024)
Supplement: Supplementary_materials_tpag024 [file supplementary_materials_tpag024.docx]

# Supporting information

Table S1: Mean growth and photosynthesis parameter estimates with 95% confidence inter- vals for the six species.

| Parameter | Units | Species | Mean | Lower 95% CI | Upper 95% CI |
| --- | --- | --- | --- | --- | --- |
| PDGR | mm yr*−*1 | ACANIG | 21.56 | 21.41 | 21.73 |
| PDGR | mm yr*−*1 | CASABB | 26.42 | 26.27 | 26.59 |
| PDGR | mm yr*−*1 | COMAPI | 25.67 | 25.51 | 25.83 |
| PDGR | mm yr*−*1 | DICCIN | 23.91 | 23.76 | 24.06 |
| PDGR | mm yr*−*1 | SCLBIR | 29.79 | 29.63 | 29.97 |
| PDGR | mm yr*−*1 | TERSER | 34.29 | 34.14 | 34.45 |
| ADI | mm | ACANIG | 5.89 | 5.70 | 6.04 |
| ADI | mm | CASABB | 6.07 | 5.88 | 6.23 |
| ADI | mm | COMAPI | 5.16 | 4.97 | 5.31 |
| ADI | mm | DICCIN | 5.61 | 5.43 | 5.75 |
| ADI | mm | SCLBIR | 7.14 | 6.94 | 7.30 |
| ADI | mm | TERSER | 9.83 | 9.65 | 9.98 |
| DGSL | days | ACANIG | 75.45 | 61.13 | 89.51 |
| DGSL | days | CASABB | 67.36 | 52.29 | 80.87 |
| DGSL | days | COMAPI | 53.26 | 39.00 | 67.06 |
| DGSL | days | DICCIN | 71.33 | 56.93 | 84.92 |
| DGSL | days | SCLBIR | 66.06 | 51.00 | 80.65 |
| DGSL | days | TERSER | 79.88 | 65.61 | 93.38 |
| *Vc max* | *µ*mol m*−*2 s*−*1 | ACANIG | 120.70 | 107.41 | 133.48 |
| *Vc max* | *µ*mol m*−*2 s*−*1 | CASABB | 113.73 | 100.41 | 126.49 |
| *Vc max* | *µ*mol m*−*2 s*−*1 | COMAPI | 124.93 | 111.79 | 137.54 |
| *Vc max* | *µ*mol m*−*2 s*−*1 | DICCIN | 121.93 | 108.38 | 134.72 |
| *Vc max* | *µ*mol m*−*2 s*−*1 | SCLBIR | 114.57 | 101.37 | 127.25 |
| *Vc max* | *µ*mol m*−*2 s*−*1 | TERSER | 150.34 | 136.62 | 163.53 |
| *Jmax* | *µ*mol m*−*2 s*−*1 | ACANIG | 135.58 | 131.42 | 139.70 |
| *Jmax* | *µ*mol m*−*2 s*−*1 | CASABB | 69.39 | 66.88 | 71.60 |
| *Jmax* | *µ*mol m*−*2 s*−*1 | COMAPI | 96.50 | 94.55 | 98.53 |
| *Jmax* | *µ*mol m*−*2 s*−*1 | DICCIN | 77.28 | 75.74 | 78.69 |
| *Jmax* | *µ*mol m*−*2 s*−*1 | SCLBIR | 130.78 | 125.70 | 135.45 |
| *Jmax* | *µ*mol m*−*2 s*−*1 | TERSER | 114.52 | 111.56 | 116.99 |
| *Amax* | *µ*mol m*−*2 s*−*1 | ACANIG | 14.96 | 14.55 | 15.37 |
| *Amax* | *µ*mol m*−*2 s*−*1 | CASABB | 11.30 | 10.99 | 11.62 |
| *Amax* | *µ*mol m*−*2 s*−*1 | COMAPI | 12.76 | 12.42 | 13.11 |
| *Amax* | *µ*mol m*−*2 s*−*1 | DICCIN | 10.99 | 10.64 | 11.35 |
| *Amax* | *µ*mol m*−*2 s*−*1 | SCLBIR | 12.90 | 12.56 | 13.24 |
| *Amax* | *µ*mol m*−*2 s*−*1 | TERSER | 14.45 | 14.10 | 14.82 |

Table S2: Coefficients (loadings) of each trait for each dimension of the principal component analysis, indicating how much each variable contributes to each dimension

| **Traits** | **Dimension 1** | **Dimension 2** | **Dimension 3** | **Dimension 4** | **Dimension 5** |
| --- | --- | --- | --- | --- | --- |
| cap_std | -0.35 | -0.14 | -0.21 | -0.18 | -0.12 |
| swc_br | -0.27 | -0.31 | 0.33 | 0.00 | -0.09 |
| RWC_tlp | -0.25 | 0.40 | 0.09 | 0.02 | 0.01 |
| sla | -0.01 | 0.50 | 0.07 | 0.34 | -0.34 |
| ptlp | 0.32 | -0.26 | 0.13 | -0.16 | -0.34 |
| wd | 0.34 | 0.07 | -0.29 | -0.05 | -0.57 |
| p50 | 0.30 | 0.11 | -0.17 | -0.52 | 0.16 |
| gmin | 0.13 | -0.46 | 0.33 | -0.04 | -0.11 |
| peak_dgr | -0.29 | -0.25 | -0.21 | 0.36 | 0.13 |
| annual_di | -0.33 | -0.18 | -0.23 | 0.07 | -0.55 |
| Vcmax | -0.26 | -0.11 | -0.50 | -0.34 | 0.10 |
| Js2000 | -0.26 | 0.11 | 0.48 | -0.35 | -0.12 |
| Am | -0.30 | 0.24 | 0.10 | -0.42 | -0.17 |

Table S3: Contribution of each species to the total variance of each dimension. Each value is expressed as percent for a total per dimension of 100%

| **Species** | **Dimension 1** | **Dimension 2** | **Dimension 3** | **Dimension 4** | **Dimension 5** |
| --- | --- | --- | --- | --- | --- |
| ACANIG | 0.10 | 31.37 | 7.01 | 20.16 | 10.81 |
| CASABR | 8.27 | 5.77 | 6.34 | 41.40 | 7.67 |
| COMAPI | 0.90 | 5.33 | 1.20 | 0.24 | 61.76 |
| DICCIN | 27.57 | 27.26 | 1.01 | 12.09 | 1.51 |
| SCLBIR | 5.64 | 9.67 | 45.21 | 8.62 | 0.31 |
| TERSER | 40.86 | 3.92 | 22.56 | 0.83 | 1.28 |

1.00 1.00


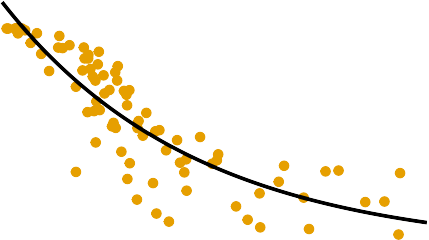


***Cassia abbreviata***


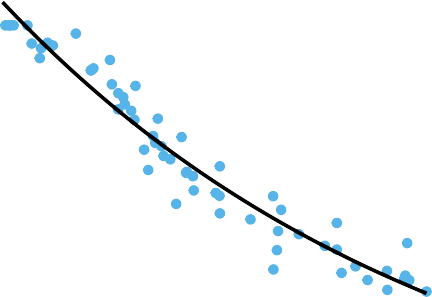


***Combretum apiculatum***

0.75 0.75

**RWC (g g^−1^)**

**RWC (g g^−1^)**

0.50 0.50

0.25

0 1 2 3 4 5 6 7 8 9 10

**Water potential (−MPa)**

0.25

0 1 2 3 4 5 6 7 8 9 10

**Water potential (−MPa)**

1.00 1.00


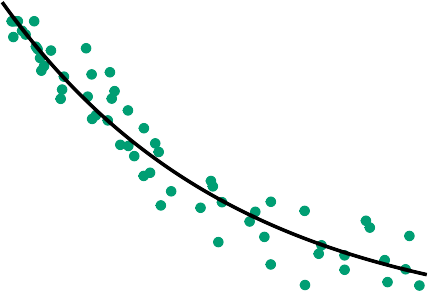


***Dichrostachys cinerea***


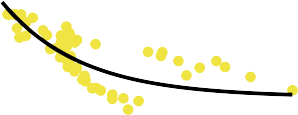


***Sclerocarya birrea***

0.75 0.75

**RWC (g g^−1^)**

**RWC (g g^−1^)**

0.50 0.50

0.25

0 1 2 3 4 5 6 7 8 9 10

**Water potential (−MPa)**

0.25

0 1 2 3 4 5 6 7 8 9 10

**Water potential (−MPa)**

1.00 1.00


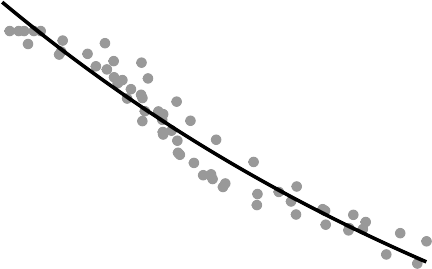


***Acacia nigrescens***


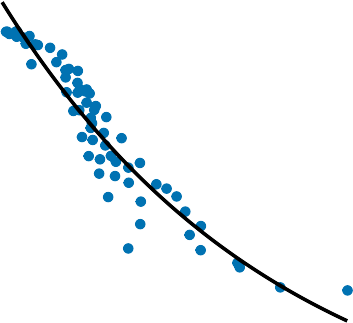


***Terminalia sericea***

0.75 0.75

**RWC (g g^−1^)**

**RWC (g g^−1^)**

0.50 0.50

0.25

0 1 2 3 4 5 6 7 8 9 10

**Water potential (−MPa)**

0.25

0 1 2 3 4 5 6 7 8 9 10

**Water potential (−MPa)**

Figure S1: Drying curves for the six species showing the relationship between RWC and leaf water potential

100 100


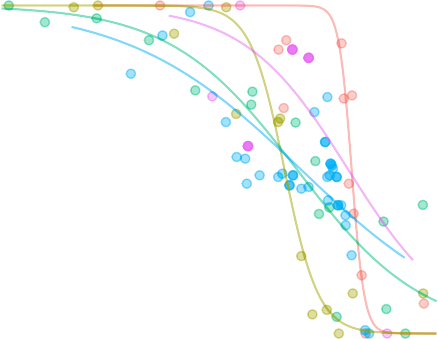


***Cassia abbreviata***


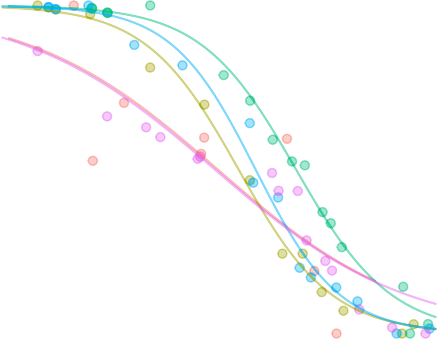


***Combretum apiculatum***

**Percent loss of air discharge (%)**

**Percent loss of air discharge (%)**

75 75

50 50

25 25

0

−12−11−10 −9 −8 −7 −6 −5 −4 −3 −2 −1 0

**Water potential (MPa)**

0

−12−11−10 −9 −8 −7 −6 −5 −4 −3 −2 −1 0

**Water potential (MPa)**

100 100


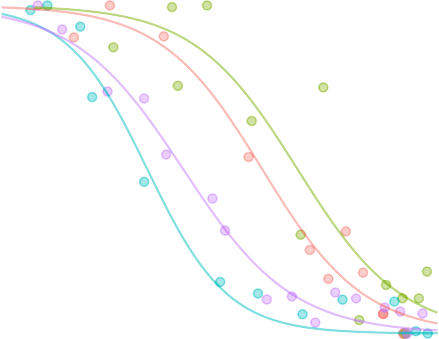


***Dichrostachys cinerea***


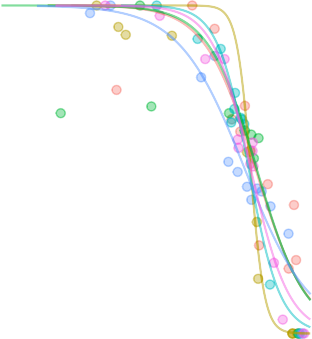


***Sclerocarya birrea***

**Percent loss of air discharge (%)**

**Percent loss of air discharge (%)**

75 75

50 50

25 25

0

100

**Percent loss of air discharge (%)**

−12−11−10 −9 −8 −7 −6 −5 −4 −3 −2 −1 0

**Water potential (MPa)**

0

100


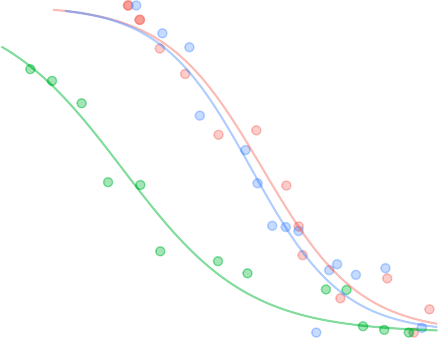


***Acacia nigrescens***

**Percent loss of air discharge (%)**

−12−11−10 −9 −8 −7 −6 −5 −4 −3 −2 −1 0

**Water potential (MPa)**

75 75


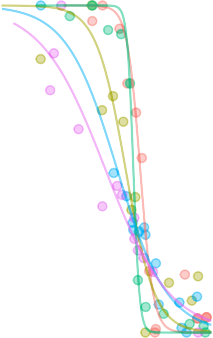


***Terminalia sericea***

50 50

25 25

0

−12−11−10 −9 −8 −7 −6 −5 −4 −3 −2 −1 0

**Water potential (MPa)**

0

−12−11−10 −9 −8 −7 −6 −5 −4 −3 −2 −1 0

**Water potential (MPa)**

Figure S2: Vulnerability curves for the six species showing the relationship between Percent loss of air discharge and xylem water potential


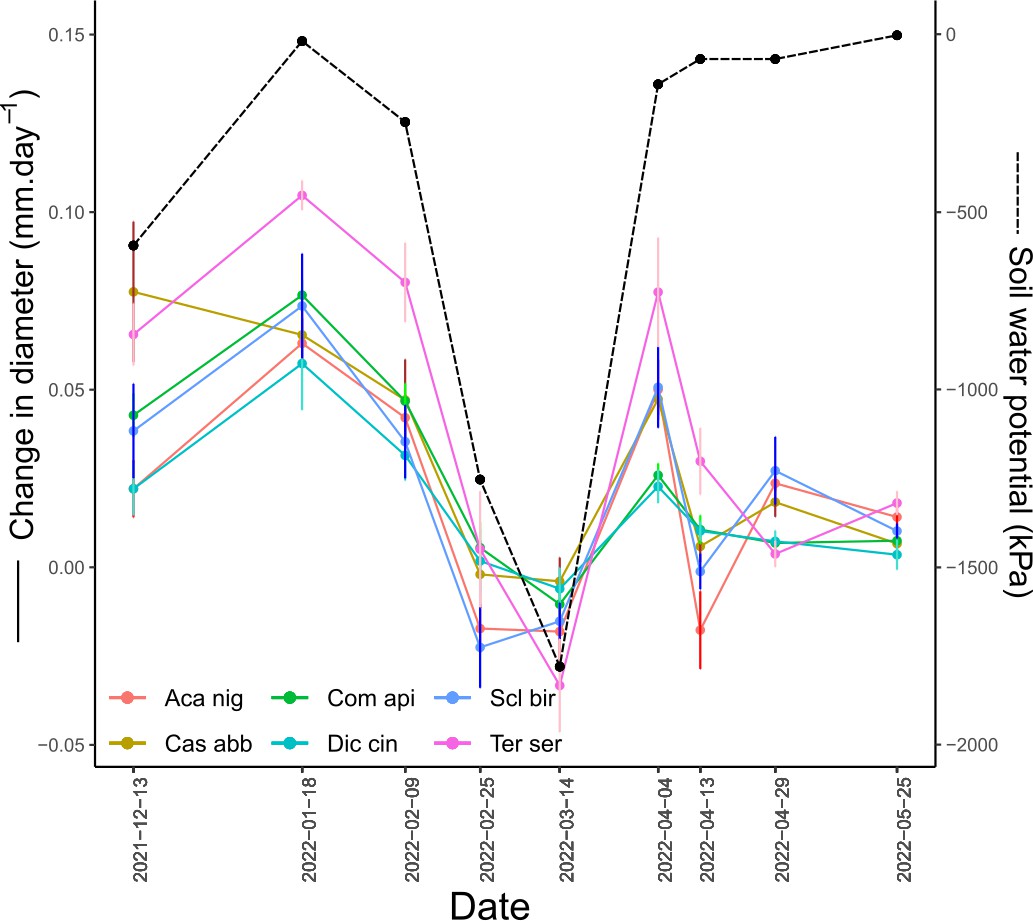


Figure S3: Growth rates of the six species for the 2021/22 rainfall year showing the decreases in growth during a growing season dry spell that occurred from the end of January until mid March 2022. The average soil water potential from three depths (15, 30 and 45 cm) is also shown.
